# Reliability and convergent validity of the Japanese version of the Adult ADHD Investigator Symptom Rating Scale: a multicenter, longitudinal, non-interventional study

**DOI:** 10.3389/fpsyt.2026.1857145

**Published:** 2026-07-28

**Authors:** Akira Iwanami, Dan Nakamura, Kensuke Ito, Nobutaka Sakayoshi, Kentaro Yamato, Tomohiro Kondo, Dorothee Oberdhan, Sayaka Machizawa, Joan Busner, Lenard A. Adler

**Affiliations:** 1 Department of Psychiatry, Showa Medical University, Tokyo, Japan; 2 Department of Clinical Science, New Drug Development Division, Otsuka Pharmaceutical Co., Ltd., Osaka, Japan; 3 Department of Clinical Science, New Drug Development Division, Otsuka Pharmaceutical Co., Ltd., Tokyo, Japan; 4 Medical Affairs Department, Otsuka Pharmaceutical Co., Ltd., Tokyo, Japan; 5 Otsuka Pharmaceutical Development and Commercialization, Inc., Global Integrated Evidence & Innovation, Rockville, MD, United States; 6 Department of Digital Health and Science, Signant Health, Blue Bell, PA, United States; 7 Department of Psychiatry, Virginia Commonwealth School of Medicine, Richmond, VA, United States; 8 Department of Science and Medicine, Signant Health, Blue Bell, PA, United States; 9 Department of Psychiatry, NYU Grossman School of Medicine, New York, NY, United States; 10 Department of Child and Adolescent Psychiatry, NYU Grossman School of Medicine, New York, NY, United States

**Keywords:** adult, AISRS-Japanese, attention deficit hyperactivity disorder (ADHD), reliability, validity

## Abstract

**Background:**

Attention deficit hyperactivity disorder (ADHD) is a neurodevelopmental disorder that often persists into adulthood, negatively impacting occupational, social, and economic outcomes. The Adult ADHD Investigator Symptom Rating Scale (AISRS) is a clinician-administered tool designed to evaluate ADHD symptom severity and treatment response. However, a validated Japanese version is not available. This study evaluated the reliability and convergent validity of the Japanese version of the AISRS (AISRS-Japanese) in adults with ADHD.

**Methods:**

Following linguistic validation procedures, the AISRS was translated into Japanese and reviewed by clinicians for conceptual and clinical equivalence. This was a multicenter, longitudinal, non-interventional study assessing the psychometric properties of the AISRS-Japanese. The evaluation instruments were the AISRS-Japanese, the Adult ADHD Self-Report Scale (ASRS-Japanese version), and the Clinical Global Impressions-Severity (CGI-S) scale. Reliability assessments included internal consistency (Cronbach’s alpha), test-retest, intra-rater, and inter-rater reliability (intraclass correlation coefficient [ICC] and G(q,k) coefficients). Convergent validity was evaluated via Pearson’s correlation between AISRS-Japanese and ASRS-Japanese version scores.

**Results:**

In total, 61 adults with ADHD were included in the analysis. The mean age of the study participants was 34 years, 52.5% (32/61) were male, and 75.4% (46/61) were moderately ill (CGI-S score of 4). The AISRS-Japanese demonstrated internal consistency for the total scale (Cronbach’s alpha = 0.83) and the hyperactivity/impulsivity and inattention subscales (0.76 and 0.75, respectively). Test-retest reliability was good (ICC: total, 0.77; hyperactivity/impulsivity, 0.79; inattention, 0.83), as was intra-rater reliability (ICC: total, 0.76; hyperactivity/impulsivity, 0.77; inattention, 0.83). Inter-rater reliability was excellent (G(q,k): total, 0.96; hyperactivity/impulsivity, 0.97; inattention, 0.96). Convergent validity of the ASRS-Japanese version was high (correlation coefficient: total, 0.89; hyperactivity/impulsivity, 0.88; inattention, 0.84).

**Conclusion:**

These results provide evidence supporting the reliability and convergent validity of AISRS-Japanese as a semi-structured tool for assessing ADHD symptoms in Japanese adults.

## 1 Introduction

Attention deficit hyperactivity disorder (ADHD) is a neurodevelopmental disorder characterized by inattention, hyperactivity, and impulsivity that occurs most frequently in childhood and adolescence (1). Currently, ADHD is diagnosed using the Diagnostic and Statistical Manual of Mental Disorders, Fifth Edition, Text Revision (DSM-5-TR) diagnostic criteria established by the American Psychiatric Association (2, 3). ADHD is a chronic condition, with symptoms often persisting into adulthood (4). The global prevalence rate of adult ADHD is 2.8% (5); in the US, it is estimated to range between 2.5% and 4.4% (6, 7); and in Japan, it is estimated to be 2.09% (8).

ADHD in adulthood has been suggested to have a negative impact across various aspects of life. Concerning occupational functioning, individuals with ADHD often experience lower employment rates, higher risk of layoffs, frequent job changes, and higher absenteeism (9–12). Regarding social functioning, ADHD has been linked to economic hardship (loss of income), higher incidence of traffic accidents, difficulty maintaining relationships leading to divorce or remaining unmarried, emotional isolation, antisocial behavior, increased mortality, and higher suicide rates (9–18). Additionally, ADHD is associated with increased medical costs (19, 20). However, appropriate treatment interventions can improve these adverse outcomes (21, 22).

The Adult ADHD Investigator Symptom Rating Scale (AISRS) is an 18-item semi-structured, clinician-administered scale that evaluates symptoms of adult ADHD in accordance with the DSM-5 (3). Each symptom is assessed through prompts that reflect adult-specific contexts, allowing clinicians to rate the severity of symptoms based on patients’ responses. The AISRS has been used as the primary endpoint measure in multiple clinical trials (23–28). Since no Japanese version of the AISRS had previously been available, the scale had not been utilized in research conducted in Japan. Therefore, we developed a Japanese version of the AISRS and conducted an evaluation of its reliability and convergent validity.

This study aimed to evaluate the reliability and convergent validity of the Japanese version of the AISRS (AISRS-Japanese) in adults with ADHD.

## 2 Materials and methods

Prior to evaluating the reliability and convergent validity of the AISRS, formal permission was obtained from the copyright holder of the AISRS to develop a Japanese version. The translation was conducted based on internationally recognized linguistic validation procedures (29). As part of the linguistic validation, the original scale was forward-translated into Japanese by a translator experienced in psychological scale translation, followed by a back-translation conducted by an independent translator. The back-translated version was reviewed by one of the scale’s original developers, to ensure conceptual equivalence with the original scale. Additionally, a clinical review was conducted by native Japanese-speaking clinicians, in consultation with the developer, to assess the appropriateness of the items from a clinical perspective. Finally, linguistic validity was verified through cognitive debriefing, and the Japanese version of the AISRS was finalized.

This longitudinal, non-interventional study was conducted at two hospitals from 20 June 2023 to 23 August 2024. The study design is shown in **Supplementary Figure 1**. The index date was defined as the date of the first study visit (Visit 1) during the patient selection period. Participants were followed for three visits, from Visit 1 until the end of the study period (**Supplementary Figure 1A**). Visit 2 was conducted within 37 days after Visit 1, and Visit 3 was conducted 14–37 days after Visit 2. However, if the primary clinician (Primary Rater) and the study participant completed all Visit 2 scales at Visit 1, only two visits were required (**Supplementary Figure 1B**). In such cases, Visit 2 took place 14–37 days after Visit 1. The follow-up period comprised the period from Visit 1 to the last visit (Visit 2 or 3) during the study. The number of follow-up days was calculated excluding the study visit date.

The AISRS-Japanese was completed by trained Primary and Sub-raters through semi-structured interviews with the study participants, and the assessment results were recorded using paper-based data collection instruments. Raters were trained on AISRS-Japanese administration and scoring via online didactic training and scoring exercises. First, raters reviewed didactic materials on scale guidance and conventions, then viewed a mock interview to practice scoring. Finally, they scored a standardized mock patient video and submitted their scores. Raters whose scores fell outside the acceptable ranges received tutorial remediation and were asked to resubmit their scores. Inter-rater reliability was assessed using AISRS-Japanese scores recorded separately by the Primary Rater and Sub-Rater at the same visit. The Primary Rater conducted a single interview, which the Sub-Rater attended; both raters rated the AISRS-Japanese based on the same interview content. To examine test-retest reliability and intra-rater reliability, the Primary Rater assessed the same participants twice at different time points. The Adult ADHD Self-Report Scale (ASRS), an established self-reported measure with a validated Japanese translation (ASRS-Japanese version), was administered to study participants as a paper-based questionnaire. The Clinical Global Impressions-Severity scale (CGI-S) was rated by the Primary Rater.

The protocol was approved by the ethics committee at Showa Medical University (approval number: 2023-178-A). The study was conducted in accordance with the Declaration of Helsinki and adhered to the Ethical Guidelines for Medical and Biological Research Involving Human Subjects.

### 2.1 Study participants

Patients who met all the following criteria were included in this study: Individuals aged 18–55 years at the time of informed consent with a diagnosis of primary ADHD based on Conners’ Adult ADHD Diagnostic Interview for DSM-IV and confirmed clinically according to DSM-5 criteria, CGI-S score ≥3 (mild severity) at Visit 1, willing and able to provide written informed consent to participate in the study, and able to answer the questionnaire and an interview in Japanese. Participants were included regardless of their pharmacological treatment status, including individuals with ADHD whose symptoms were stable under treatment as judged by the investigators.

Exclusion criteria were as follows: Individuals participating in any clinical trial; pregnant or lactating women; individuals with symptom(s) of schizophrenia, bipolar disorder, and substance use disorder; and/or individuals with major comorbid psychiatric illnesses other than schizophrenia, bipolar disorder, and active substance use disorder, as judged by the investigators.

### 2.2 Rating scales

The evaluation instruments included the AISRS-Japanese, ASRS-Japanese version, and CGI-S. All evaluators received standardized training using common materials prior to the initiation of the study for the AISRS-Japanese and CGI-S assessments. As part of the qualification process, AISRS-Japanese raters scored a standardized mock patient interview video to establish scoring consensus.

The AISRS-Japanese assesses symptoms of ADHD in adult patients using a semi-structured interview methodology with suggested prompts to contextualize each symptom to adult situations. It includes 18 items that directly correspond to the symptoms of ADHD. The AISRS-Japanese includes hyperactivity/impulsivity and the inattention subscales, each consisting of nine items. Each of the 18 items is rated on a 4-point scale with scores from 0 (none) to 3 (severe), resulting in a total score ranging from 0–54 points (0–27 points for subscales), where higher scores indicate more severe symptoms of ADHD (30).

The ASRS-Japanese version is a self-report instrument with established reliability and validity, designed to assess subjects’ perceptions of the 18 DSM-defined symptoms of adult ADHD. The ASRS-Japanese version includes hyperactivity-impulsivity and inattention subscales, and each subscale consists of nine items. Symptom severity is evaluated based on the reported frequency of each item. Each item is rated on a 5-point scale from 0 (never) to 4 (very often), resulting in a total score ranging from 0–72 points (0–36 points for subscales), where higher scores indicate more severe symptoms of ADHD (31).

The CGI-S is a single-item clinician-rated measure used to assess overall disease severity, in this case ADHD. It is scored on a 7-point scale, considering symptom severity, frequency, associated distress, and the impact on behavior and functioning. CGI-S scores are1 “normal, not at all ill”, 2 “borderline mentally ill”, 3 “mildly ill”, 4 “moderately ill”, 5 “markedly ill”, 6 “severely ill”, and 7 “among the most extremely ill” (32).

### 2.3 Statistical methods

The target sample size was set at 60 participants based on the Consensus-based Standards for the Selection of Health Measurement Instruments checklist, which considers a sample size of ≥50 very good and 30–49 participants adequate for assessing construct validity (33). The primary analysis population used in this study was the intention-to-treat (ITT) set, which included study participants who completed all baseline and follow-up visits with/without some missing data on the instruments, and the per-protocol (PP) set, which included all study participants who completed all baseline and follow-up visits without any missing data. As this study aimed to evaluate the convergent validity and reliability of the AISRS-Japanese, both ITT and PP set analyses were conducted to ensure a more accurate assessment of the scale’s psychometric properties, with the latter restricted to participants with consistent responses and reliable data.

All statistical procedures were pre-specified. Descriptive statistics were used to summarize the demographic and clinical characteristics of the study participants, including mean ± standard deviation (SD) and median (interquartile range) for continuous data and n (%) for categorical data.

The internal consistency of AISRS-Japanese was assessed using Cronbach’s alpha, which measures the average correlation of items within a scale (34) for the total scale and each subscale. The cut-off was 0.7, with ≥0.7 indicating an acceptable internal consistency for the total scale or subscales of the AISRS-Japanese (34).

The test-retest reliability assesses the variation in measurements taken by an instrument on the same subject under the same condition (35). This assessment was restricted to participants with stable symptoms, defined as no discrepancy between the CGI-S scores at Visit 2 and Visit 3 (or Visit 1 and Visit 2, if all scales planned for Visit 2 were collected at Visit 1). We evaluated this by calculating the intraclass correlation coefficient (ICC) for the total scale and subscales of the AISRS-Japanese. ICC values were calculated using a two-way mixed-effects model, single-measure estimates, and an absolute agreement definition. ICC values <0.50 indicated poor reliability; 0.50–0.75, moderate reliability; 0.75–0.90, good reliability; and >0.90, excellent reliability (35).

The intra-rater reliability of the AISRS-Japanese (which assesses the variation of data when the same rater assesses the same participant across ≥2 occasions (35)) was evaluated by calculating the ICC and its 95% confidence interval (CI) for the total scale or subscales of the AISRS-Japanese rated by the Primary Rater at Visit 2 and Visit 3 (or Visit 1 and Visit 2). The intra-rater reliability assessment was performed on all participants, regardless of the stability of their ADHD symptoms.

The inter-rater reliability of the AISRS-Japanese (which assesses the variation between ≥2 raters who measure the same group of subjects (35)) was assessed for the total scale and subscales based on the G(q,k) equation and its 95% CI, using the AISRS-Japanese scores from the Primary Rater and Sub-rater, both of which were collected at the same visit. Values <0.50 indicate poor reliability; 0.50–0.75, moderate reliability; 0.75–0.90, good reliability; and >0.90, excellent reliability.

Pearson’s correlation coefficient was used to assess convergent validity. Specifically, we assessed: (a) correlation between concurrently collected AISRS-Japanese total score and ASRS-Japanese version total score (rated by study participants) at Visit 2 (or Visit 1, if all scales planned for Visit 2 were collected at Visit 1), and (b) correlation between concurrently collected AISRS-Japanese subscale scores (rated by Primary Raters) and ASRS-Japanese version subscale scores (rated by the study participants) at Visit 2 (or Visit 1, if all scales planned for Visit 2 were collected at Visit 1). Considering that both instruments assess ADHD symptom domains, we hypothesized that the AISRS-Japanese total and subscale scores would show positive correlations with the corresponding ASRS-Japanese scores. An absolute value of 0.4 in the AISRS-Japanese was used as the cut-off, with ≥0.4 supporting convergent validity and <0.4 indicating low convergent validity (30).

A single missing ASRS value was imputed with the mean score for all other subscale items, and the data were analyzed only within the ITT set. All statistical analyses were performed using SAS software version 9.2 or higher (SAS Institute Inc., Cary, NC, USA) and R software version 4.2.3 or higher (R Foundation for Statistical Computing, Vienna, Austria).

## 3 Results

### 3.1 Study participants

**Figure 1:**
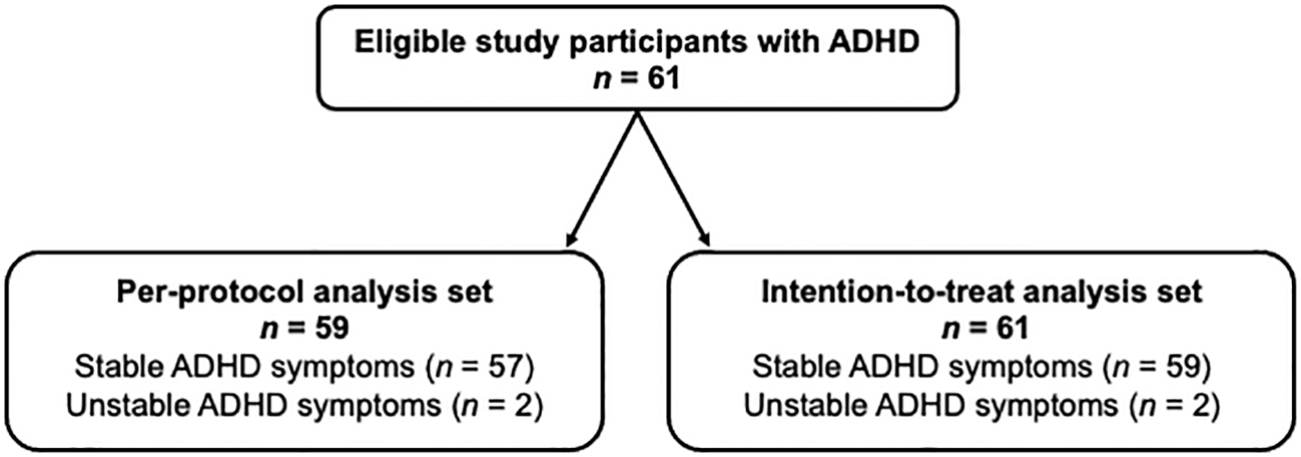
Study participant flow chart. ADHD, attention deficit hyperactivity disorder.

The participant flow chart is shown in **Figure 1**. In total, 61 and 59 participants met the eligibility criteria and were included in the ITT set and PP set, respectively.

The demographic and clinical characteristics of the study participants are shown in **Table 1**. In the ITT set, the mean age of the study participants was 34.0 (SD: 10.9) years, and 52.5% (32/61) were male. Most participants (75.4%, 46/61) were moderately ill (CGI-S score of 4). Regarding the ADHD subtype, 55.7% (34/61) had the inattentive subtype, and 44.3% (27/61) had the combined subtype. Only 9.8% (6/61) of the study participants had visited a clinician for ADHD during childhood. There were no notable differences in demographic and clinical characteristics between the ITT and PP sets.

**Table 1:** Demographic and clinical characteristics.

Characteristics	ITT set *N* = 61	PP set *N* = 59
Age, years		
Mean ± SD	34.0 ± 10.9	33.6 ± 10.8
Median (IQR)	32.0 (24.0–44.0)	32.0 (24.0–44.0)
Sex, n (%)		
Female	29 (47.5)	27 (45.8)
Male	32 (52.5)	32 (54.2)
History of clinician visits for ADHD during childhood, n (%)	6 (9.8)	6 (10.2)
ADHD subtype		
Inattentive	34 (55.7)	33 (55.9)
Hyperactive/impulsive	0 (0.0)	0 (0.0)
Combined	27 (44.3)	26 (44.1)
CGI-S score, n (%)		
1 (normal, not at all ill)	0 (0.0)	0 (0.0)
2 (borderline ill)	0 (0.0)	0 (0.0)
3 (mildly ill)	9 (14.8)	9 (15.3)
4 (moderately ill)	46 (75.4)	45 (76.3)
5 (markedly ill)	5 (8.2)	4 (6.8)
6 (severely ill)	1 (1.6)	1 (1.7)
7 (most extremely ill)	0 (0.0)	0 (0.0)

ADHD, attention deficit hyperactivity disorder; CGI-S, Clinical Global Impressions-Severity; IQR, interquartile range; ITT, intention-to-treat; PP, per-protocol; SD, standard deviation.

### 3.2 Internal consistency of AISRS-Japanese total scale and subscales

Internal consistency of the AISRS-Japanese total scale in the ITT and PP sets is shown in **Table 2**. In the ITT set, the corrected item-total correlations indicated that most AISRS-Japanese items were considered appropriate for the instrument. Hartley’s Fmax was 1.79, indicating the variance of the items in the AISRS-Japanese total scale was homogeneous. Cronbach’s alpha was 0.83, indicating acceptable internal consistency. Results in the PP set were consistent with those in the ITT set.

**Table 2:** Internal consistency of the AISRS-Japanese total scale and subscales (ITT and PP sets).

AISRS-Japanese Scale/Sub-scale	ITT set		PP set	
	Item-variance difference	Cronbach’s alpha	Item-variance difference	Cronbach’s alpha
	F_max_		F_max_	
Total scale	1.79	0.83	1.85	0.82
Hyperactivity/impulsivity subscale	1.70	0.76	1.80	0.74
Inattention subscale	1.67	0.75	1.68	0.75

AISRS-Japanese, Japanese version of the Adult ADHD Investigator Symptom Rating Scale; ITT, intention-to-treat; PP, per-protocol.

Internal consistency of the AISRS-Japanese subscales in the ITT sets is also shown in **Table 2**. The corrected item-total correlations indicated that most AISRS-Japanese items were considered appropriate for the instrument. Hartley’s Fmax was 1.70 in the hyperactivity/impulsivity subscale and 1.67 in the inattention subscale, indicating that variances of the items in each AISRS-Japanese subscale were homogeneous. Cronbach’s alpha was 0.76 in the hyperactivity/impulsivity subscale and 0.75 in the inattention subscale, indicating acceptable internal consistency. Results in the PP set were consistent with those in the ITT set (**Table 2**).

### 3.3 Test-retest, intra-rater, and inter-rater reliability of the AISRS-Japanese

The results of test-retest reliability and intra-rater reliability of the AISRS-Japanese in the ITT set are shown in **Table 3**. In the test-retest reliability assessment, the point values of ICC for the hyperactivity/impulsivity subscale, inattention subscale, and total scale were 0.79, 0.83, and 0.77, respectively, indicating good test-retest reliability. In the intra-rater reliability assessment, the point values of ICC for the hyperactivity/impulsivity subscale, inattention subscale, and total scale were 0.77, 0.83, and 0.76, respectively, indicating good intra-rater reliability. In the inter-rater reliability assessment, the point values of G(q,k) for the hyperactivity/impulsivity subscale, inattention subscale, and total scale were 0.97, 0.96, and 0.96, respectively, indicating excellent inter-rater reliability. Similar results were shown in the PP set (**Table 3**).

**Table 3:** Test-retest reliability and intra-rater reliability of the AISRS-Japanese (ITT and PP sets).

AISRS-Japanese	Test-retest reliability		Intra-rater reliability		Inter-rater reliability	
	ICC	95% CI	ICC	95% CI	G (q,k)	95% CI
ITT set						
Hyperactivity/impulsivity subscale	0.79	0.67–0.87	0.77	0.64–0.86	0.97	0.95–0.98
Inattention subscale	0.83	0.73–0.90	0.83	0.72–0.89	0.96	0.94–0.98
Total scale	0.77	0.64–0.86	0.76	0.63–0.85	0.96	0.93–0.98
PP set						
Hyperactivity/impulsivity subscale	0.79	0.66–0.87	0.76	0.63–0.85	0.97	0.94–0.98
Inattention subscale	0.84	0.74–0.90	0.83	0.73–0.90	0.97	0.95–0.98
Total scale	0.77	0.64–0.86	0.76	0.62–0.85	0.96	0.93–0.97

AISRS-Japanese, Japanese version of the Adult ADHD Investigator Symptom Rating Scale; CI, confidence interval; ICC, intraclass correlation coefficient; ITT, intention-to-treat; PP, per-protocol.

### 3.4 Convergent validity of the AISRS-Japanese

In the ITT set, the correlation coefficient between the AISRS-Japanese and ASRS total scores was 0.89, indicating a high convergent validity. In addition, strong convergent validity was supported in both subscale scores of the AISRS-Japanese with each counterpart of the ASRS (correlation coefficient: 0.88 for the hyperactivity/impulsivity subscale and 0.84 for the inattention subscale). In the PP set, the respective correlation coefficients were consistent with those for the ITT set (0.88, 0.87, and 0.83, respectively).

## 4 Discussion

The present study is the first to assess the reliability and convergent validity of the AISRS-Japanese. The study findings provide evidence supporting the reliability and convergent validity of the AISRS-Japanese for assessing ADHD symptoms in Japanese adults, reinforcing its potential clinical usefulness in Japanese adults with ADHD. Further studies are needed to evaluate its responsiveness to treatment and utility for monitoring treatment-induced symptom changes in clinical practice and clinical trials.

Internal consistency, test-retest, intra-rater, and inter-rater reliability were assessed in the present study to evaluate the reliability of the AISRS-Japanese. The results demonstrated that measures were within acceptable ranges, confirming the reliability of the AISRS-Japanese. Cronbach’s alpha coefficients for internal consistency were 0.83 for the AISRS-Japanese total scale, 0.76 for the hyperactivity/impulsivity subscale, and 0.75 for the inattention subscale. These values all exceeded the pre-specified threshold of 0.70 for acceptable internal consistency (36), demonstrating good overall reliability for the total scale and acceptable consistency for the individual subscales, thereby supporting that each item on the AISRS-Japanese was measured consistently and that the scale can adequately assess adult ADHD symptoms. In addition, the ICC of the test-retest reliability over an interval of 14–37 days showed high stability, with values of 0.77 for the total scale, 0.79 for the hyperactivity/impulsivity subscale, and 0.83 for the inattention subscale. These results confirm that the AISRS-Japanese is a reliable tool for assessing ADHD symptoms consistently over time. As for inter-rater reliability, the G(q,k) coefficient was extremely high at 0.96 for the total scale, 0.97 for the hyperactivity/impulsivity subscale, and 0.96 for the inattention subscale. Inter-rater reliability was assessed based on a single interview conducted by the Primary Rater and attended by the Sub-rater. Thus, these coefficients should be interpreted as reflecting scoring agreement based on shared interview content rather than agreement across fully independent administrations of the interview. Nonetheless, this indicates that the AISRS-Japanese maintains high reproducibility and consistency across different raters and is a reliable instrument for use by multiple healthcare professionals in clinical settings. Similarly, for intra-rater reliability, the ICC was as high as 0.76 for the total scale, 0.77 for the hyperactivity/impulsivity subscale, and 0.83 for the inattention subscale, indicating that consistent results were obtained with repeated measurements by the same rater. Notably, these reliability results are highly consistent with findings from a previous study on the original English version of the AISRS, where Cronbach’s alpha was 0.80 for the total score, 0.79 for the inattention subscale, and 0.76 for the hyperactivity/impulsivity subscale, and test-retest ICCs ranged from 0.66 to 0.78 (37). This consistency underscores that the Japanese version demonstrates reliability equivalent to the original instrument. The results are also consistent with a previous study that reported Cronbach’s alpha values of 0.82–0.83 at baseline and 0.95 at 6 months for the total score; 0.74–0.75 at baseline and 0.94 at 6 months for the inattention subscale; and 0.85–0.86 at baseline and 0.90 at 6 months for the hyperactivity/impulsivity subscale, with test-retest reliability showing a concordance correlation coefficient of 0.90 (30).

To evaluate validity, convergent validity was assessed. The AISRS-Japanese total score showed a very high correlation coefficient of 0.89 with the ASRS-Japanese version, and the hyperactivity/impulsivity and inattention subscale scores also showed high correlation coefficients of 0.88 and 0.84, respectively, with the ASRS-Japanese version. These results suggest that the AISRS-Japanese, similar to the ASRS-Japanese version, is a valid tool for assessing adult ADHD symptoms.

### 4.1 Limitations

The present study has some limitations, including those inherent to the longitudinal study design. This non-interventional study employed a short-term longitudinal design, with test-retest evaluations conducted at two time points with intervals ranging from 14–37 days. This test-retest interval was deliberately selected to reduce recall or practice effects, while maintaining logistical feasibility within a multicenter non-interventional, routine clinical environment. Owing to the limited sample size, a sensitivity analysis stratified by the exact length of the test-retest interval was not performed. Therefore, this interval variability is an important factor to consider when evaluating our test-retest reliability estimates. In addition, the design was not sufficient to capture symptom changes resulting from treatment initiation or modification, or to assess long-term outcomes. A more rigorous psychometric evaluation using the larger phase 3 trial dataset is planned to further examine additional measurement properties, including structural validity, responsiveness, and clinically meaningful change. The present study did not assess structural validity. Although the AISRS includes theoretically meaningful inattention and hyperactivity/impulsivity subscales, exploratory or confirmatory factor analysis was not conducted because the limited sample size did not meet the recommended participant-to-item ratio for structural testing. In addition, ADHD diagnoses were established using the Conners’ Adult ADHD Diagnostic Interview, based on DSM-IV criteria. Investigators applied DSM-5 criteria clinically rather than using a structured DSM-5-validated diagnostic instrument. Although this approach reflects routine clinical practice, the use of a fully DSM-5-validated diagnostic scale may have improved diagnostic precision. The study population consisted of adults with relatively stable ADHD symptoms. Therefore, the generalizability of the findings to more severe cases or individuals with comorbid conditions may be limited. Another limitation is that most participants were moderately ill, and none of them had the predominantly hyperactive/impulsive subtype. Therefore, the study findings may not be generalizable to patients with a presentation of hyperactivity/impulsivity or those with significant comorbidities. Further studies are needed to explore the applicability of the AISRS-Japanese in populations with more diverse clinical profiles. Additionally, measurement errors were not evaluated. Other aspects of validity, including discriminant validity and known-groups validity, were not examined, which may affect the overall evaluation of the measure. Specifically, because this study did not include comparisons between the AISRS-Japanese and measures of major comorbid symptoms, such as depression or anxiety, discriminant validity could not be evaluated within the scope of this study.

## 5 Conclusion

Among Japanese adults with ADHD, the AISRS-Japanese was shown to provide a convergent and reliable measure of ADHD symptoms, supporting its potential utility in clinical practice and research. Further studies are needed to evaluate its responsiveness to treatment and utility for monitoring symptom changes before and after treatment. Given the semi-structured format of the AISRS-Japanese, consistent and accurate administration requires that clinicians receive adequate training.

## Data Availability

The datasets presented in this article are not readily available due to the sensitive nature of the research and because participants did not provide written consent for public release of their data. Requests to access the datasets should be directed to Tomohiro Kondo, Kondo.Tomohiro.a@otsuka.jp.
