# Supplementary material for: Reliability and convergent validity of the Japanese version of the Adult ADHD Investigator Symptom Rating Scale: a multicenter, longitudinal, non-interventional study
Source: Front Psychiatry. 2026 Jul 28;17:1857145. doi: 10.3389/fpsyt.2026.1857145 (PMC13457393; doi:10.3389/fpsyt.2026.1857145)
Supplement: Supplementary file 1 [file DataSheet1.pdf]

# *Supplementary Material*

## Supplementary Figure

(A)

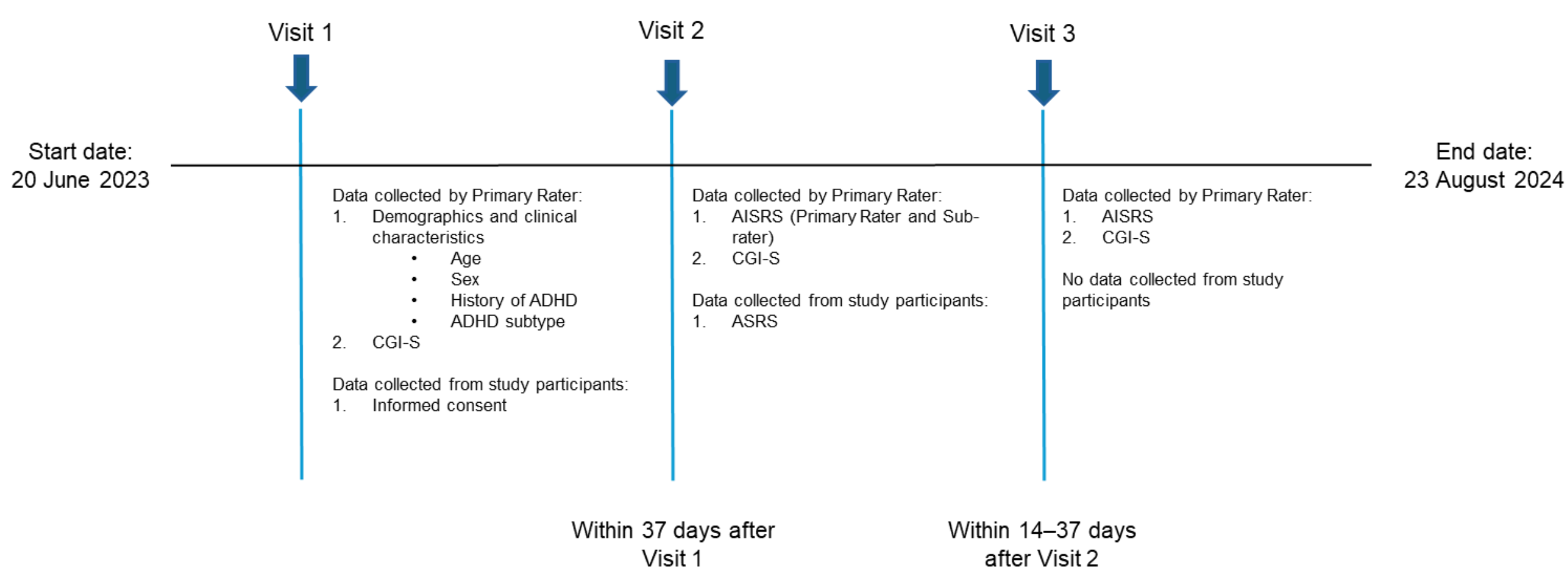


(B)

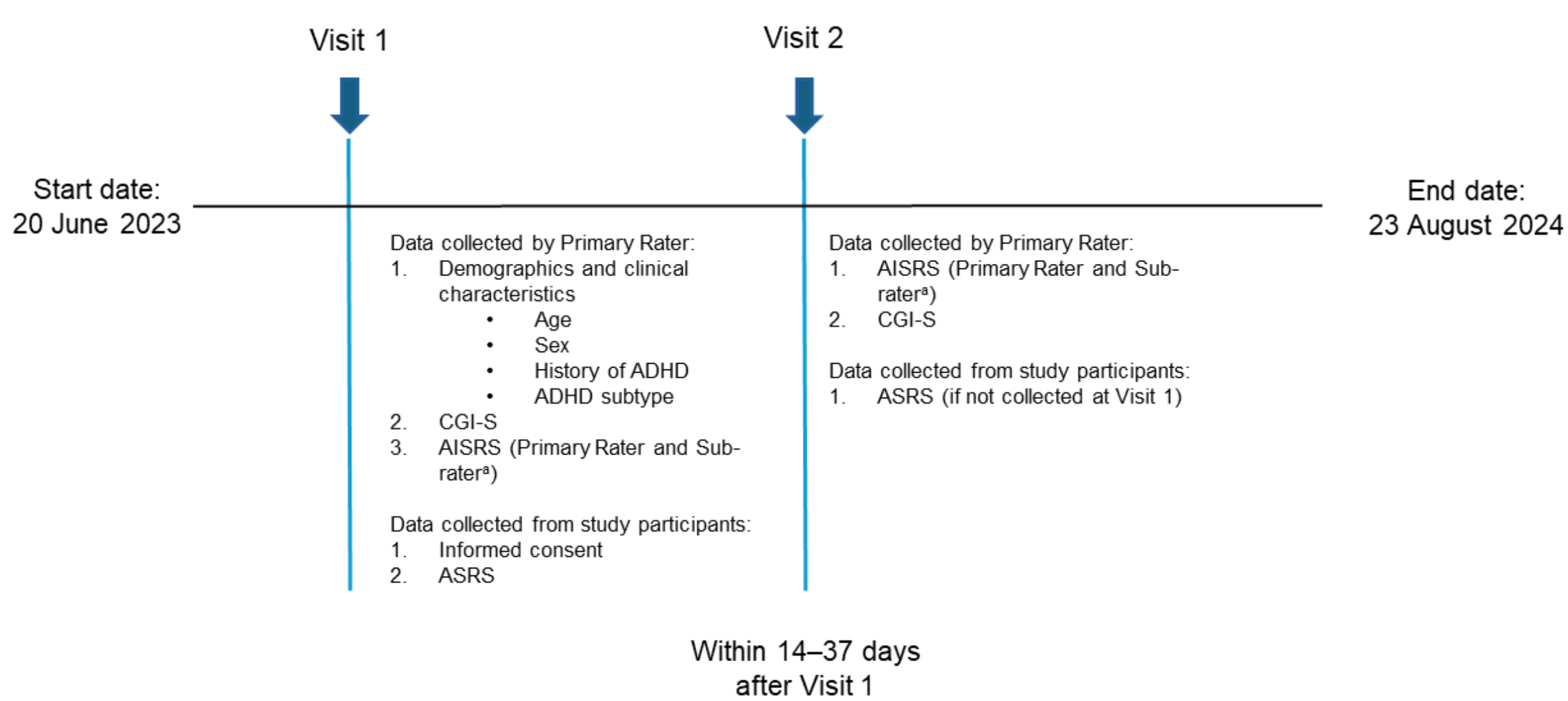


**Supplementary Figure 1.** Study design: (**A**) with three visits and (**B**) with two visits
ADHD, attention deficit hyperactivity disorder; AISRS, Adult ADHD Investigator Symptom Rating Scale; ASRS, Adult ADHD Self-Report Scale; CGI-S, Clinical Global Impressions-Severity.
